# The Dental Pulp

**Published:** 1888-04

**Authors:** Junius E. Cravens

**Affiliations:** Indianapolis, Ind.


### The Dental Pulp.

BY JUNIUS E. CRAVENS, D.D.S., INDIANAPOLIS, IND.

[Read before the Mississippi Valley Dental Association, held at Cincinnati, O., March 8, 1888.]

1st. The pulp or completed dentine papilla is the first anatomical element within the *sac* to assume the form of a tooth.

A deposition of calcific matter on the surface of the dentine organ (the pulp) fixes the character of the *crown* to be developed.

The enamel organ is compelled to conform to the crust of dentine by the binding in force of the fibrous sac-wall.

Magitot evidently observed carefully upon certain points but simply guessed at others or accepted without question that which he found ready written upon points he did not feel inclined to investigate. Otherwise I can not account for his statement that the enamel organ determines or fixes the form of the crown of the tooth. Dean's translation of Magitot's "Origin of the Dental Follicle" contains numerous errors.

2d. By virtue of fibrils (soft fibres of Tomes) that are projected from the odontoblasts, the pulp presides over or promotes the tubular or canalicular formation of dentine. The odontoblastic fibrils act first as *mandrels*, around which calcific matter is deposited.

3d. After a slight shell of dentine has been formed upon the surface of the pulp, the further development of dentine *in* the crown is accomplished solely through the agency of the pulp.

When the development of the root of a tooth has progressed to near the proper or destined terminus of dentine, and the walls of the pulp chamber are of proper thickness for strength, etc., a special activity of odontoblasts upon one or more sides of the entrance to the root canal is manifested, resulting in a partial constriction of the canal by development of secondary dentine at the entrance —a condition often noted by practitioners of dentistry.

This constriction evidently is provided for the purpose of reducing the vital force of the pulp within the crown by partial strangulation of that organ, thus reducing the odontoblasts within the chamber to a state of at least temporary inactivity, and preventing further general deposit of salts of dentine upon the walls of the pulp chamber.

4th. Calcification, as described, at the entrance of a root canal, if not interfered with, will eventually so strangle the pulp as to cause its death. An attempt to provide against such a fatal contingency is made by subsequent calcification at and beyond the apical terminus of dentine, by which strangulation is again applied to reduce the vital powers of the pulp, so that the former constrictive calcification is arrested at the entrance to the root canal; and the pulp may thenceforth act only as a central source of supply for surrounding dental tissue.

5th. As the apex of a root is always completed by cementum, the apical construction is caused by development of cementum; never by dentine.

The apical constriction is the indicator by which the operator is warned that he has explored the canal far enough. That which lies beyond the apical constriction is a sacred temple, designated by Prof. G. V. Black the "apical space," to enter which is disastrous.

6th. If constriction calcification at the ends of the root canal be not performed, then secondary dentine may be developed within the pulp chamber; or, calcification of the pulp itself may progress until the organ dies. Pulp "nodules" or "stones" are also manifestations of a tendency of the pulp to persistent calcification.

7th. It would appear, then, that persistent calcification is the final function of the dental pulp. If so, to what extent is the preservation of the pulp essential to the welfare of a completed tooth in the human subject?

---

ACETONEMIA—The peculiar chloroform-like odor of the breath, so commonly noticeable in diabetes, is due to the presence of ethyl-diacetate, formerly called acetone, in the blood. It is also observed in some cases of gastric cancer, gastritis and alcoholism, irrespective of diabetic symptoms.

